# Modification of Glucose Metabolic Pathway to Enhance Polyhydroxyalkanoate Synthesis in *Pseudomonas putida*

**DOI:** 10.3390/cimb46110761

**Published:** 2024-11-10

**Authors:** Yue Dong, Keyao Zhai, Yatao Li, Zhen Lv, Mengyao Zhao, Tian Gan, Yuchao Ma

**Affiliations:** 1 College of Biological Sciences and Technology, Beijing Forestry University, Beijing 100083, China; 2 College of Forestry, Beijing Forestry University, Beijing 100083, China

**Keywords:** PHAs, metabolic pathway, *Pseudomonas putida*, genetic modification, mcl-PHA production

## Abstract

Medium-chain-length polyhydroxyalkanoates (mcl-PHAs) are semi-crystalline elastomers with a low melting point and high elongation at break, allowing for a wide range of applications in domestic, agricultural, industrial, and mainly medical fields. Utilizing low-cost cellulose hydrolyzed sugar as a carbon source and metabolic engineering to enhance synthesis in *Pseudomonas putida* is a promising strategy for commercializing mcl-PHAs, but little has been attempted to improve the utilization of glucose for synthesizing mcl-PHAs. In this study, a multi-pathway modification was performed to improve the utilization of substrate glucose and the synthesis capacity of PHAs. To enhance glucose metabolism to flow to acetyl-CoA, which is an important precursor of mcl-PHA, multiple genes in glucose metabolism were inactive (branch pathway and negative regulatory) and overexpressed (positive regulatory) in this study. The two genes, *gcd* (encoding glucose dehydrogenase) and *gltA* (encoding citrate synthase), involved in glucose peripheral pathways and TCA cycles were separately and jointly knocked out in *Pseudomonas putida* QSRZ6 (*ΔphaZΔhsdR*), and the mcl-PHA synthesis was improved in the mutants; particularly, the mcl-PHA titer of QSRZ603 (*ΔgcdΔgltA*) was increased by 33.7%. Based on the glucose branch pathway truncation, mcl-PHA synthesis was further improved with *hexR*-inactivation (encoding a negative regulator in glucose metabolism). Compared with QSRZ603 and QSRZ6, the mcl-PHA titer of QSRZ607 (*ΔgcdΔgltAΔhexR*) was increased by 62.8% and 117.5%, respectively. The mutant QSRZ609 was constructed by replacing the endogenous promoter of *gltB* encoding a transcriptional activator of the two-component regulatory system GltR/GltS with the ribosome subunit promoter P_33_. The final mcl-PHA content and titers of QSRZ609 reached 57.3 wt% and 2.5 g/L, an increase of and 20.9% and 27.3% over that of the parent strain QSRZ605 and an increase of 110.4% and 159.9% higher as compared to QSRZ6, respectively. The fermentation was optimized with a feeding medium in shaker flacks; then, the mcl-PHA contents and titer of QSRZ609 were 59.1 wt% and 6.8 g/L, respectively. The results suggest that the regulation from glucose to acetyl-CoA by polygenic modification is an effective strategy for enhancing mcl-PHA synthesis, and the mutants obtained in this study can be used as chassis to further increase mcl-PHA production.

## 1. Introduction

It is expected that global plastic production will reach 540 million metric tons by 2040 [1,2], and about 12 billion metric tons of plastic waste would accumulate in landfills or the natural environment by 2050, as synthetic polymers do not biodegrade [3,4]. With the increasing scarcity of fossil resources and the intensification of environmental problems, the development and utilization of bio-based degradable and high-performance plastics are of growing interest [5]. Polyhydroxyalkanoates (PHAs) are bio-polyesters intracellularly accumulated by a variety of bacteria as a storage material of carbon and energy, they are reduced under unfavorable growth conditions in the presence of an excess carbon source, and they are fully degradable in soil, lake water, seawater, sewage, sludge, and even aerobic and anaerobic conditions [6]. PHAs have been used as potential alternatives to petroleum-based plastics due to their unique properties such as biodegradability, biocompatibility, excellent thermal and mechanical properties, and diverse material properties suitable for industrial and medical applications [7].

PHAs, as aliphatic polyesters well known for safely biodegrading in any environment, are synthesized by *Bacillus megaterium*, *Ralstonia eutropha*, *Cupriavidus necator*, and *Pseudomonas putida*, along with others from the genera *Azotobacter*, *Syntrophomonas*, *Aeromonas*, and *Clostridium* [8]. Several bacteria have been employed for the industrial production of PHAs, including *Alcaligenes latus* and *Ralstonia eutropha* for scl-PHAs, *R. eutropha* and *Aeromonas hydrophila* for PHBHHx, and *Pseudomonas* spp. for mcl-PHA [9]. However, the major limitations of the currently produced PHAs, especially short-chain PHAs, are their poor mechanical properties and their high production costs [9]. While the mechanical properties can be improved by producing mcl-PHAs and polymer blending, the high production cost is of greater concern [10]. The current cost of producing PHAs is USD 1.68 per kilogram, which by far exceeds that of petroleum-based plastics [9]. Currently, PHAs are produced from the fermentation of sugars and fatty acids extracted from food crops, such as corn starch, sugarcane, and vegetable oil. The feedstock cost itself accounts for more than 50% of the production cost. To combat this, by utilizing abundant and low- or zero-cost lignocellulosic biomass as a carbon source, chassis bacteria were multi-pathway-modified along with integrating PHA production into biorefinery processes [11].

In contrast to scl-PHAs, mcl-PHAs possess very different mechanical properties, including a relatively low decomposition temperature, a low degree of crystallinity, strong elasticity, a low glass transition temperature, and high elongation at break [12,13]. Due to these improved properties, mcl-PHAs are promising materials in various fields, especially in biomedical applications, such as tissue engineering and drug delivery [14,15,16]. The biosynthesis of mcl-PHA by *Pseudomonas* spp. with related carbon sources (e.g., fatty acids) is achieved via the β-oxidation pathway, but metabolized substrates indirectly correlated with carbon sources (e.g., glucose and glycerol) are also achieved via the fatty acid de novo synthesis pathway [17]. In previous studies, various molecular strategies such as medium optimization and genetic manipulation were explored to increase mcl-PHA production, including the engineering of expression promoters [18,19], large-scale deletion of genomic islands [20] and the block of the formation of by-products [21], and improvements in the precursor synthesis [22] and morphology engineering [23]. However, the production cost of PHAs is still higher than that of the petrochemical plastics for many reasons, such as expensive substrate, low production limited by biosynthetic efficiency and cell size, and difficult downstream processing. It may be an effective way to improve PHA synthesis efficiency by metabolic engineering with low-cost indirectly related carbon sources [24]. So, improving the PHA biosynthesis capacity and the utilization of indirectly related carbon sources of strain is a technical bottleneck that must be solved in the commercial production of PHAs by means of metabolic engineering.

In *Pseudomonas* spp., glucose is converted into acetyl-CoA through the EDEMP cycle; then, it enters the fatty acid de novo synthesis pathway (FAS), and, finally, it is further transformed into PHA through the pha gene cluster [25]. The level of PHA synthesis with glucose as an indirect carbon source is largely determined by the ability of glucose to be converted to acetyl-CoA. Glucose enters into the periplasmic space through OprB, and it is either transported to the cytosol or converted into gluconate. Glucose in the periplasmic can also be transported directly to the cytoplasm through an ABC uptake system, and the first acting enzyme is glucokinase (Glk), which phosphorylates glucose to give glucose 6-phosphate (G6P). Next, the combined action of glucose 6-phosphate dehydrogenase (Zwf) and 6-phosphogluconolactonase (Pgl) converts G6P into 6-phosphogluconate (6PG) [26]. The produced 6PG enters to the Entner–Doudoroff route and is converted into 2-keto-3deoxy-6-phosphogluconate (KDPG) by the action of the 6-phosphogluconate dehydratase (Edd) and then hydrolyzed to produce glyceraldehyde-3-phosphate and pyruvate by the action of the KDPG aldolase (Eda) [27]. Glyceraldehyde-3-phosphate is further metabolized by the glyceraldehyde-3-phosphate dehydrogenase (Gap-1) to D-glycerate 1,3-bisphosphate, while pyruvate is decarboxylated to acetyl-CoA and enters the Krebs cycle. The genes involved in the glucose metabolism in *Pseudomonas* are arranged in several operons, and the genes’ expression is controlled by the concerted action of HexR and GltR/GtrS [28].

**Figure 1:**
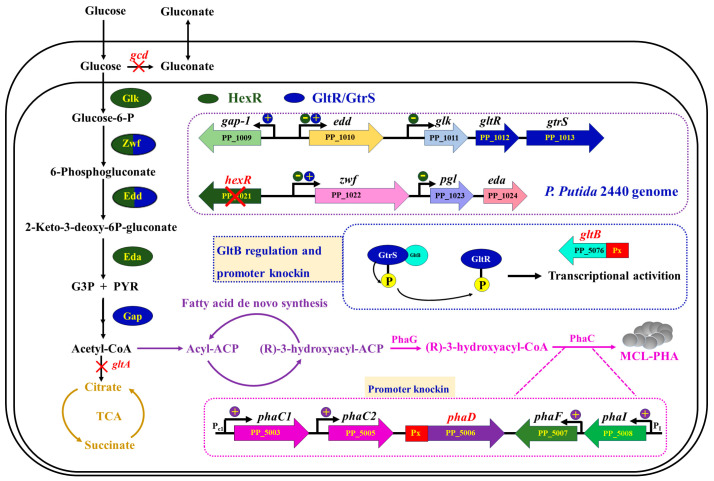
A combination strategy of multiple genes’ modification for improving the mcl-PHA yield in *P. putida* QRSZ6. The modified genes are marked in red.

*Pseudomonas putida* KT2440, an ideal mcl-PHA production chassis from glucose, possesses a clear genetic background, relatively definite cellular metabolic networks, and natural genetic manipulation systems to be further engineered for efficient production of mcl-PHA [29,30]. In this study, mutant *P. putida* QSRZ6 (*ΔphaZΔhsdR*) constructed in our lab from *P. putida* KT2440 was used to improve the conversion efficiency from glucose to acetyl-CoA. The key genes (*gcd* and *gltA*) of two branched metabolic pathways were disrupted individually and coupled by a homologous recombination double-exchange, and several regulators were knockout (*hexR*) or enhanced expression (*gltB* and *phaD*) by a strong promoter replacement. A schematic diagram of the overall strategy for enhancing mcl-PHA production in *P. putida* QSRZ6 is shown in Figure 1. Finally, the fermentation conditions of the modified mutants were optimized.

## 2. Materials and Methods

### 2.1. Strains and Plasmids

*Escherichia coli* DH5α and S17-1 were used for plasmid construction and conjugation, respectively. *P. putida* QSRZ6, as the mutant of *P. putida* KT2440 with inactive *phaZ* (encoding the PHA degrading enzyme) and *hsdR* (encoding Type I restriction modification system endonuclease), was elected as the initial strain for genetic modification. The derivatives of pK18*mobsacB* were used to construct responding marker-less gene deletion, promoter knock-in, and gene insertion. All strains and plasmids used in this study are listed in Table 1.

### 2.2. The Construction of Recombinant Plasmids and Mutants

All primers in this study were designed with primers 5.0 and are listed in Table S1. Using genomic DNA as a template, DNA fragments were PCR-amplified with a corresponding primer set (Table S1). Purified PCR products, as upstream and downstream homology arms for gene-inactivation or promoter knock-in and gene for overexpression, were inserted into pK18*mobsacB* at the site of *Eco*R I/*Hin*d III by Gibson assembly to construct recombinant vectors. The reconstructed plasmids were transformed into *E. coli* S17-1 to generate donor cells for the later biparental conjugation with *P. putida* (Figure S1A). Homologous recombination single and double crossover mutants were screened on YT plates (per L: 10 g yeast extract, 20 g tryptone, and 14 g agar) supplemented with kanamycin (50 μg/mL) and 100 g/L sucrose, respectively. The mutants were corroborated by colony PCR amplification with the upstream primers of the upstream homologous arm and the downstream primers of the downstream homologous arm as primer pairs (Figure S1B–J) and sanger sequencing by Sangon Biotech Co., Ltd. (Shanghai, China).

### 2.3. Culture Conditions

*E. coli* and *P. putida* were cultivated in a Luria–Bertani (LB) medium at 37 °C and 30 °C, respectively. If necessary, kanamycin (Km, 50 μg/mL) or/and ampicillin (Amp, 50 μg/mL) was used as a resistance marker in the medium for maintenance of plasmids or inhibition of donor bacteria. To prepare cell suspension, single colonies of *P. putida* strains were picked form the plate and inoculated into a 50 mL flask containing 10 mL LB and then incubated at 30 °C and 225 rpm for 12 h. By taking a calculated volume of the obtained cell suspension from the pre-culture (to begin the PHA-accumulating process with an initial OD_600_ of 0.2), 500 mL shake flasks containing 50 mL low-nitrogen M9G medium were inoculated and placed in a rotary shaker under aerobic conditions at 30 °C and 225 rpm. The low-nitrogen M9G medium contained 15 g glucose, 12.8 g Na_2_HPO_4_·7H_2_O, 3 g KH_2_PO_4_, 1 g (NH_4_)_2_SO_4_, 1 g yeast extract, 0.5 g NaCl, 0.12 g MgSO_4_·7H_2_O, and 250 μL trace element solution per liter. The trace element solution per liter contained 2.4 g FeSO_4_·7H_2_O, 1.08 g CaCO_3_, 0.8 g ZnSO_4_·H_2_O, 0.464 g MnSO_4_·H_2_O, 0.148 g CoSO_4_·7H_2_O, 0.132 g CuSO_4_·5H_2_O, and 0.032 g H_3_BO_3_. Cell growth was recorded as optical density (OD) at 600 nm on a Microplate reader (Infinite^®^ 200PRO, TECAN, Männedorf, Switzerland). The cell dry weight (CDW) was determined gravimetrically after the collection of a 50 mL culture broth for 10 min at 10,000 rpm through a refrigerated centrifuge (Avanti J-26S XP, Beckman, Brea, CA, USA) in pre-weighed tubes, including a washing step with distilled water, and by drying the obtained pellet at 100 °C until attaining a constant weight. The glucose concentration in the cultivation supernatant and medium was analyzed by the 3,5-dinitrosalicylic acid method at 540 nm after appropriate dilution. Three independent replicates were performed for each culture sample.

### 2.4. RT-qPCR

For mRNA analysis, the cell pellet was harvested at 8 h and 26 h from aliquots of 1 mL fermentation broth. The RNAprep Pure cultured cell/bacterial total RNA extraction kit was chosen for total RNA extraction. The 1% agarose gel electrophoresis and Nanodrop (NanoPhotometer^®^ NP80, Implen, Munich, Germany) were used for integrity analysis and concentration determination. The cDNA was synthesized according to the protocol of FastKing cDNA First Strand Synthesis Kit (TIANGEN, KR116, Beijing, China). With 16S rRNA gene as the internal reference and about 100 ng/μL cDNA as the template, CFX ConnectTM Optics Module (Bio Rad Laboratories, Inc., Hercules, CA, USA) was chosen to carry out qPCR according to the instruction of TB Green ^®^ Premix EX Taq TM (Tli RNaseH Plus; Takara, RR420A, Beijing, China) using methods described previously [31]. The comparative CT method (2^−∆∆Ct^) was performed to determine the levels of endogenous genes expressed [32].

### 2.5. PHA Extraction, Characterization, and Quantification

PHA composition of the polymer produced, as well as the cellular PHA content concentration, were determined by gas chromatography and mass spectrometry (Shimadzu, GCMS-QP2010SE, Shanghai, China), and the software GCMS solution 2.6. PHA was extracted and methylated by the method described by Poblete-Castro et al. [21], and methyl esters of monomers were analyzed by GC-MS. An aliquot (1 μL) of the organic phase was injected into the gas chromatograph at a split ratio of 1:30. Separation of the esterified monomers of interest was achieved by an Aglilent DB-1 capillary column (30 m × 0.25 mm i.d. × 0.25 film thickness). Nitrogen was used as the carrier gas at a flow rate of 0.8 mL/min. The injector and transfer line temperature were 200 °C and 320 °C, respectively. The oven temperature program started with an initial temperature 80 °C for 1 min, then from 80 °C up 120 °C at a rate of 10 °C/min, and finally up to 160 °C for 5 min at a rate of 45 °C/min. The PHA content (wt%) was defined as the percentage of the CDW represented by the polyhydroxyalkanoate.

### 2.6. Optimization of Fermentation Conditions

The fermentation conditions of the mutants were optimized in shake flasks, including initial medium type, initial glucose concentration, feeding strategy, and pH regulation. Firstly, LB, LBG (add 20 g glucose to LB liquid medium), high-nitrogen M9GI (with 2.5 g/L ammonium sulfate and 2.5 g/L yeast extract), M9GII (with 5 g/L yeast extract), and M9GIII (with 5 g/L ammonium) were used to culture bacteria, and the cell concentration was measured by optical density of OD_600_ at 2, 3, 4, 5, 6, 8, 12, 18, and 24 h of culture, respectively. Secondly, the initial glucose concentration (10, 20, 30, 40, 50 g/L) was optimized according to the cell growth and residual glucose content at 2, 3, 4, 5, 6, 8, 12, 18, and 24 h during fermentation. Thirdly, two feeding strategies were carried out to improve the PHA synthesis. One was to take a one-time glucose supplement to 10 g/L by adding a feed solution (500 g/L glucose and 10 g/L MgSO_4_·7H_2_O) at 24 h. Another was to replenish glucose to 4–6 g/L by adding the same feed solution when glucose is reduced to 2 g/L with the multiple small supplements. The cell dry weight, PHA content, and titer were measured at 48 h, 62 h, and 74 h, respectively.

### 2.7. Statistical Analysis

Three independent replicates were performed for each culture sample. Data with error bars represent the means and standard deviations. One-way ANOVA and Duncan’s test were used to analyze whether there were significant differences between multiple treatment groups through SPSS 25. Origin 2021 was used to visualize data.

## 3. Results

### 3.1. The Interdicting of the Competing Branch Pathway in Glucose Metabolism

**Figure 2:**
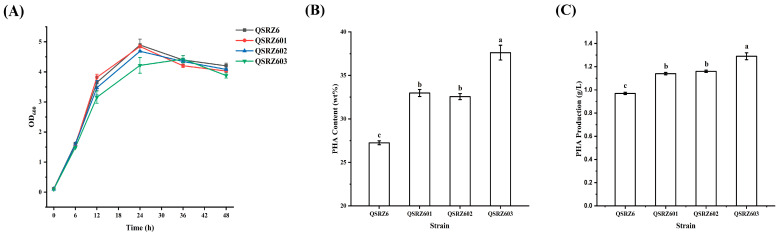
The cell growth (**A**), mcl-PHA content (**B**), and production (**C**) of *gcd* and *gltA* gene knockout mutants. Letters indicate significant differences (*p* < 0.05; One-way ANOVA; Duncan’s test) between strains.

The single and double gene knockout mutants of *gcd* (encoding glucose dehydrogenase) and *gltA* (encoding citrate synthase) were constructed by homology recombination double exchange with antibiotics markerless based on *phaZ* (PHA hydrolase) mutant *P. putida* QSRZ6 (Figure S1A,B). The culture profile and glucose consumption of the mutants on 20 g/L glucose as the only carbon and energy source in batch formation are given in Figure 2A and Figure S2A. The cell growth rate and glucose consumption rate of the single gene knockout mutant QSRZ601 (*Δgcd*) and QSRZ602 (*ΔgltA*) were similar to those of the parent strain QSRZ6. But those of the double gene inactivation (QSRZ603, *ΔgcdΔgltA*) slightly lowered down. The mutants revealed a much higher synthesis of the desired polymer (Figure 2B,C). The mcl-PHA content of QSRZ601, QSRZ602, and QSRZ603 reached 33.0, 32.6, and 37.6 wt%, respectively. Compared to the QSRZ6 (27.3 wt%), which was enhanced by 21.0%, 19.5%, and 38.0%, respectively. The final mcl-PHA titers of QSRZ601, QSRZ602, and QSRZ603 were 1.1, 1.2, and 1.3 g/L, thus 18.2, 19.7, and 33.7% higher as compared to the parent strain (1.0 g/L). The two branch pathways were truncated, which obviously supported the redirection of carbon to anabolism and mcl-PHA biosynthesis.

### 3.2. The Inactive of Transcriptional Negative Regulatory Factors in Glucose Metabolism

**Figure 3:**
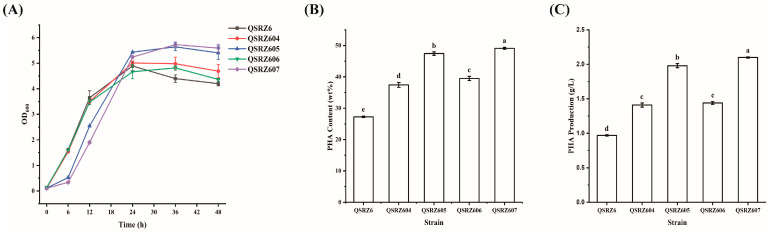
The cell growth (**A**), mcl-PHA content (**B**), and production (**C**) of the *hexR*-inactive mutants. Letters indicate significant differences (*p* < 0.05; One-way ANOVA; Duncan’s test) between strains.

To relieve the inhibition of HexR and enhance the glucose metabolic rate for PHA synthesis, the *hexR*-inactive mutants QSRZ604 (*ΔhexR*), QSRZ605 (*ΔhexRΔgcd*), QSRZ606 (*ΔhexRΔgltA*), and QSRZ607 (*ΔhexRΔgcdΔgltA*) were constructed from the parent strain QSRZ6, QRZ601, QRZ602, and QRZ603, respectively. Compared to the corresponding parent strain, the growth rate of the mutants was lower within 24 h but obviously higher from 24 h to 48 h, and more cells were obtained at 48 h except QSRZ606, which had similar levels to its parent (Figure 3A). However, the glucose consumption rates of the multiple gene inactive mutants QSRZ605 and QSRZ607 significantly slowed down, and the residual glucose reached zero at about 36 h (Figure S2B). The content of the mutants QSRZ604, QSRZ605, QSRZ606, and QSRZ607 reached 37.4 wt%, 47.4 wt%, 39.5 wt%, and 49.1 wt%, an increase of 37.4%, 43.8%, 21.3%, and 30.6% over that of their corresponding parent strain; an increase of 37.4%, 74.0%, 45.0%, and 80.2% over that of QSRZ6. The final mcl-PHA titers of the modified strains QSRZ604, QSRZ605, QSRZ606, and QSRZ607 were 1.4 g/L, 2.0 g/L, 1.4 g/L, and 2.1 g/L, thus 46.0%, 73.7%, 24.1%, and 62.8% higher as compared to the corresponding parent strain; 45.5%, 104.7%, 48.3%, and 117.5% higher as compared to QSRZ6 (Figure 2B,C). The results revealed *hexR*-inactive was important for mcl-PHA synthesis, especially coupled with *gcd* and *gltA* knockout. Given that QSRZ605 and QSRZ607 have similar final mcl-PHA titers, QSRZ605 with a faster growth rate and glucose utilization rate was selected for further genetic modification.

### 3.3. The Enhancement of Transcriptional Positive Regulatory Factors in Glucose Metabolism

**Figure 4:**
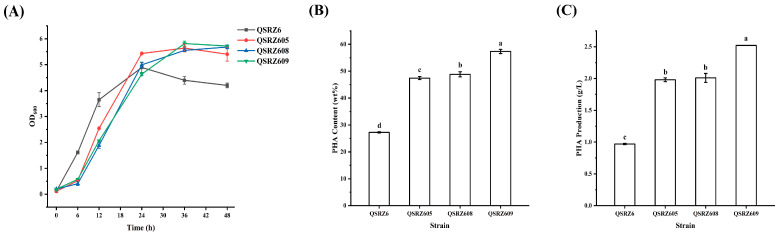
The cell growth (**A**), mcl-PHA content (**B**), and production (**C**) of the *gltB* overexpression mutants. Letters indicate significant differences (*p* < 0.05; One-way ANOVA; Duncan’s test) between strains.

To improve the efficiency of glucose catabolism through GtrS-GltR signaling cascade, the expression of *gltB* was enhanced by the promoter knock-in method with the strong promoters of ribosomal small subunit protein S1 and ribosomal regulator (P_17_ and P_33_) from *P. putida* KT2440 reported by Zhang et al. [11]. The growth and glucose consumption rates in the mutants QSRZ608 (QSRZ605::P_17_-*gltB*) and QSRZ609 (QSRZ605::P_33_-*gltB*) were similar to those in the parent strain QSRZ605 (Figure 4A and Figure S3C). The mcl-PHA content and final titer of the mutant QSRZ608 did not increase significantly. However, the mcl-PHA content and final titer of QSRZ609 reached 57.3 wt% and 2.5 g/L, an increase of and 21.0% and 27.3% over that of their corresponding parent strain; an increase of 110.4% and 159.9% higher as compared to QSRZ6, respectively (Figure 4B,C). The results suggested that P_33_ was more suitable for *gltB* overexpression intensity, which enhanced the mcl-PHA synthesis through the GtrS-GltR signaling cascade.

### 3.4. Modification of P. putida KT2440 PHA Synthesis Pathway

**Figure 5:**
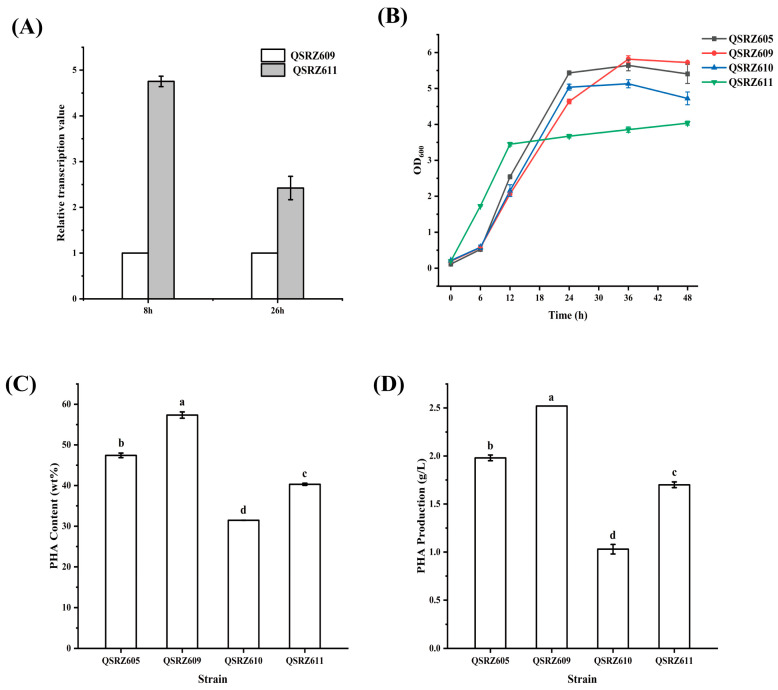
Characteristics of *phaD* gene overexpression mutants. Transcription levels of *phaD* detected by qRT-PCR (**A**); the cell growth (**B**); the mcl-PHA content (**C**); and production (**D**). Letters indicate significant differences (*p* < 0.05; One-way ANOVA; Duncan’s test) between strains.

To enhance the expression of mcl-PHA syntheses, the *phaD* gene controlled by the promoter P_33_ and P_17_ was inserted inside the *hexR* gene, and the mutants QSRZ610 (QSRZ605::P_33_-*phaD*) and QSRZ611 (QSRZ609::P_33_-*phaD*) were constructed with QSRZ605 and QSRZ609 as parents, respectively. The relative expression of the *phaD* gene in QSRZ610 and QSRZ611 was significantly higher than that in their responding parent strain (Figure 5A). However, the results surprisingly showed that the final cell dry weight, mcl-PHA content, and titer were all lower than their respective parent strains (Figure 5A,B). The glucose consumption rate of QSRZ611 was not faster than that of QSRZ609, but that of QSRZ610 returned to a similar level to that of QSRZ6 and was faster than QSRZ605 (Figure S3A). The mcl-PHA content and final titer of QSRZ610 were 33.7% and 48.0% lower than those of the parent strain QSRZ605, respectively. The PHA content and final titer of QSRZ611 were 29.7% and 32.5% lower than those of the parent strain QSRZ609 (Figure 5C,D), respectively. It suggests that PhaD may regulate other unknown carbon metabolic pathways, which will require further study.

### 3.5. PHA Structural Analysis

The main monomers of mcl-PHAs were C10 (3HD), followed by C8 (3HO), C12 (3HDD), and C14 (3HTD), while the content of C6 (3HHx) was extremely low with glucose as an unrelated carbon source in *P. putida* KT2440 [31]. The PHA monomers of QSRZ6 were detected using GC/MS, and it was found that the proportion of each monomer was similar to what was previously reported (Table 2). The PHA monomer type was determined by the de novo synthesis of fatty acids and the function of PHA synthase in the PHA biosynthesis with glucose as a substrate. The type and ratio of PHA monomer from the mutants-only modified glucose metabolic pathway in this study were consistent with those from the starting strain QSRZ6 (Table 2), which was as expected.

### 3.6. Optimization of Fermentation Condition

**Figure 6:**
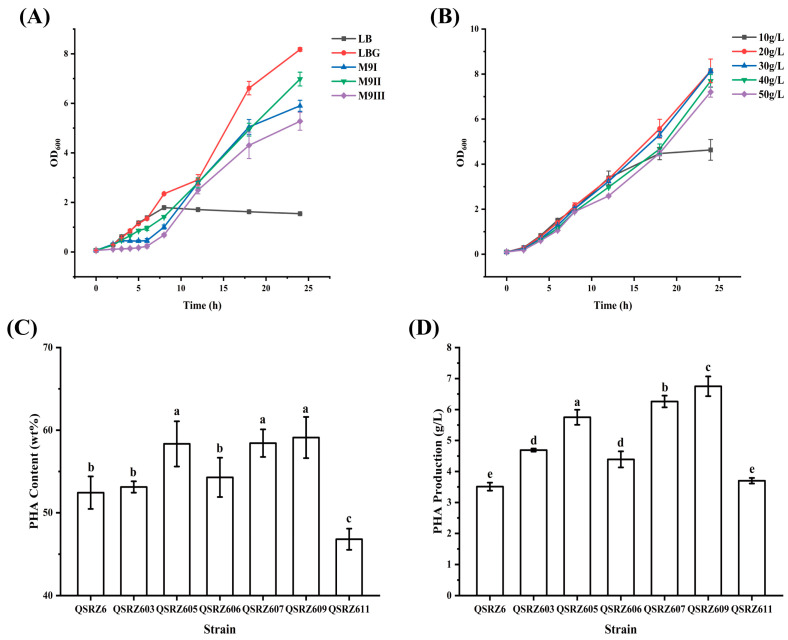
The optimization of fermentation conditions. The cell growth in different mediums (**A**) and in LBG with different concentrations of glucose (**B**); the mcl-PHA content (**C**) and production (**D**) of the modified mutants in an optimized medium. Letters indicate significant differences (*p* < 0.05; One-way ANOVA; Duncan’s test) between strains.

To select the appropriate initial medium, five different mediums including LB, LBG (with 20 g/L glucose), high-nitrogen M9GI (with 2.5 g/L ammonium sulfate and 2.5 g/L yeast extract), M9GII (with 5 g/L yeast extract), and M9GIII (with 5 g/L ammonium) were used to culture QSRZ607. The highest OD_600_ value was obtained in LBG at 24 h, which was selected to be the most suitable medium for cell growth (Figure 6A). To obtain the maximum number of cells and avoid the waste of carbon sources, different concentrations of glucose were added to the LB medium for culture QSRZ607. The highest CDW, mcl-PHA content, and production were obtained with 30 g/L glucose (Table S2). However, a lot of glucose residue was found with high-concentration initial glucose (30–50 g/L), the lower OD_600_ value was found with 10 g/L initial glucose, and the optimal glucose concentration was 20 g/L with the highest OD_600_ value and relatively little residual glucose at for 24 h (Figure 6B and Figure S3A). The cell growth curve was not affected by pH adjustments (Figure S3B). In order to increase the synthesis of mcl-PHA, two feeding strategies were adopted. One was to take a one-time glucose supplement to 10 g/L at 24 h. Another was to replenish glucose to 4–6 g/L when glucose was reduced by 2 g/L with multiple small supplements during 24–72 h (Figure S3C). The results showed that the final titers with the two strategies had no significant differences at fermentation at 48 h. Although the titers of 62 and 74 h of small multiple feeding fermentation were slightly higher than those of 48 h of single feeding (Table S3), considering the cost, the single feeding was more appropriate.

Finally, the optimized conditions were used for the feeding fermentation of the three excellent mutants including QSRZ605, QSRZ607, and QSRZ609, and the production levels of CDW and mcl-PHAs of each strain were further detected (Figure 6C,D). The highest mcl-PHA content in the feeding fermentation culture was 59.1 wt% (QSRZ609), followed by 58.4 wt% (QSRZ607) and 53.1 wt% (QSRZ605). The highest mcl-PHA titer reached 6.8 g/L (QSRZ609), which was then followed by 6.3 g/L (QSRZ607) and 5.8 g/L (QSRZ605), increasing yields by 92.6%, 78.4%, and 63.9% compared with QSRZ6, respectively. In conclusion, QSRZ609 was an excellent mutant strain, which produced 0.2 g mcl-PHA per gram of glucose.

## 4. Discussion

Plastics have become ubiquitous materials essential for modern life and sustaining the global economy. Almost 368 million tons of plastics are consumed each year [33]. However, if the current growth in the use of plastics continues, the plastics sector will account for 20% of total fossil consumption by 2050 [34]. Growing scarcity and the rising cost of raw materials and serious concerns around climate change from fossil fuels have firmly placed the manufacture of plastics based on renewable raw materials back on the center stage. PHAs are the only known bioplastics that are entirely produced and safely degraded by living cells in any environment. However, the major limitations of the currently produced PHAs, especially short-chain PHAs, are their poor mechanical properties and their high production costs [9]. While the mechanical properties can be improved via producing mcl-PHAs and polymer blending, the high production cost is of greater concern [10,35]. However, their cost, despite nearly four decades of commercial availability, is 2–4 times that of traditional plastics [36]. Currently, PHAs are synthesized by bacteria from the fermentation of sugars and fatty acids extracted from food crops, such as corn starch, sugarcane, and vegetable oil. The feedstock cost itself accounts for more than 50% of the production cost. To combat this, cellulose hydrolyzed glucose from abundant and low- or zero-cost lignocellulosic biomass, such as agricultural residues, forest residues, agro-food processing waste, and dedicated energy crops, can be used as a carbon source for PHA production. However, several challenges exist in terms of low PHA yields, need for pre-treatment, inhibition during fermentation, and downstream recovery of PHAs [35]. Some members of genus *Pseudomonas* are well known to synthesize mcl-PHA from glucose or fatty acid. *Pseudomonas putida* KT2440, an ideal mcl-PHA production chassis from cellulose hydrolysis glucose, possesses low inhibition during fermentation, clear genetic background, relatively definite cellular metabolic networks, and natural genetic manipulation systems to be further engineered for efficient production of mcl-PHA [29,30]. *P. putida* QSZR6, a restriction modification system and PHA degradation capacity-deficient mutant from *Psedomonas putida* KT2440, was previously constructed in our lab, which was used as chassis to engineer fermentation glucose to produce mcl-PHA.

Mcl-PHA granules were first detected in *Pseudomonas oleovorans* GPo1 in1983 [37]. Since then, several members of other genus, such as *Bacillus thermonamylovorans* PHA005 [38], *Streptomyces* sp. JM3 [39], *Ralstoonia eutropha* NCIM 5149 [40], and *Cupriavidus necator* TISTR 1095 [41], have been investigated as mcl-PHA producers and as chassis to engineer enhanced mcl-PHA production. For mcl-PHA production, the carbon sources influence the cell growth rate, PHA titer, and proportion and type of monomers in the polymer [37,40]. Substrates structurally related carbon sources, including fatty acids and oils, can be metabolized via the β-oxidation pathway. Structurally unrelated carbon sources, such as sugars, volatile fatty acids, and glycerol, are converted into acetyl-CoA through glycolysis and then into mcl-PHA through the de novo fatty acid biosynthesis pathway. A higher median-specific growth rate on structurally unrelated carbon sources than related carbon sources, but mcl-PHA production, was more efficient on rare and expensive related carbon sources [37]. Most studies have used structurally related substrates as significant carbon sources for efficient production mcl-PHA. The mcl-PHA title of *P. aeruginosa* STN-10 fermentation frying oil reached 23.7 g/L [42]; those of *P. putida* KT2442 and KT2440 fermentation oleic acid [43] and nonanoic acid [44] reached 72.6 g/L and 52.5 g/L, respectively. However, mcl-PHA titles of *Bacillus subtilis* KP172548 fermentation glucose only reached 3.1 g/L [45]; those of *Ralstonia eutropha* NCIM 5149 [40] and *Cupriavidus necator* TISTR 1095 [41] fermentation starch only reached 1.1 g/L and 0.1 g/L approximately; and that of *Pseudomonas chlororaphis* DSM 19603 fermentation apple pulp only reached 4.3 g/L [46]. With approximately 50% of the production costs attributed to the fermentation substrate, a significant opportunity exists to dramatically cut the cost of PHA production through the use of a low- or no-cost substrate. Cellulose is the most abundant biopolymer on earth, and using cellulose-hydrolyzed sugar as a fermentation substrate to produce mcl-PHA is a sustainable way to reduce costs. However, in this process, the key is the efficient use of cellulose hydrolyzed sugars by bacteria to synthesize mcl-PHA. Here, with QSZR6 as chassis, several genetic modifications were performed for this purpose: we truncated the branching pathway of glucose metabolism, knocked out the gene coding for the negative regulator, and enhanced the expression of positive regulatory factors by strong promoter knock-in along with fermentation optimization.

Firstly, the branching pathways of glucose metabolism were truncated. When *P. putida* takes glucose as the only carbon source, about 90% of the glucose entering the cell from the extracellular environment mostly through the periplasmic space is converted into the para-metabolite gluconate by glucose dehydrogenase (encoded by *gcd*, PP_1444) in *P. putida* [47]. According to previous reports for *P. putida*, gluconate was detected as a major by-product [48] and accumulated in large amounts in the broth and reached a maximum level of 12 g/L after 35 h of cultivation, resulting in an overall mcl-PHA yield of 0.1 g (g glucose)-1 [21]. The final mcl-PHA titer *P. putida* KT2440 *Δgcd* was 1.8 g/L and 100% higher as compared to the parent strain [21]. Citrate synthase (encoded by *gltA*, PP_4194) is the first enzyme and catalyzes the production of citric acid with acetyl-CoA and oxaloacetate as substrates into TCA cycle. In *E. coli* that heterogeneously expressed PHA synthetic gene, *gltA*-inactive mutant decreased the growth rate but increased the mcl-PHA yield [49]. After knocking out, the cell growth and glucose utilization were basically the same as those of the original strain QSRZ6, and the CDW (3.5 g/L) of QSZ601 was not significantly different from the CDW (3.6 g/L) of the original strain QSRZ6, indicating that the gene *gcd* was not a key gene affecting cell growth, and its removal did not influence the bacterial growth or the rate of absorption and utilization of carbon sources. The content of PHA was 33.0 wt%, an increase of 21.0% compared to QSRZ6 (27.3 wt%), indicating that knocking out the *gcd* gene can increase the carbon flux flowing to the EDEMP pathway, indirectly causing more carbon sources to generate PHAs to increase the production of PHAs [21]. The TCA cycle is also an important competitive pathway for the synthesis of PHAs. After knocking out the *gltA*, the OD_600_ of cell growth was slightly reduced, but the utilization rate of glucose was not affected. The CDW of QSRZ602 had no significant difference compared with that of QSRZ6, while the content of PHA increased by 19.5%. All these results showed that truncating the TCA cycle could allow for more carbon flux to flow to the FAS pathway, which was conducive to the generation of mcl-PHAs. In addition, the deletion of both genes affected the growth rate of cells as well as the utilization of glucose. Also, CDW and PHAs were improved compared to both the original strain and the single gene mutants, proving that coupling knockout of *gcd* and *gltA* genes can play a positive role in increasing the synthetic ability of PHAs.

Secondly, the gene *hexR* coding for the negative regulator were inactive. The HexR (pp_1021) regulator belongs to the RpiR family of transcriptional regulators and acts as a repressor in the sugar catabolism of Gram-negative [50]. In Pseudomonas, the HexR regulator controls the transcription of the *zwf*/*pgl*/*eda* and *edd*/*glk*/*gltR-2* operons as well as the gap-1 gene. The HexR regulator exerts its regulatory action by binding to specific sequences (5′-TTGT-N7/8-ACAA-3′) in the target promoters, which, in turn, prevents the progress of RNA polymerase [51]. HexR recognizes 2-keto-3-deoxy-6-phosphogluconate (KDPG), an intermediate of Entner–Doudoroff pathway, and is required for the catabolism of several sugars such as glucose, fructose, and gluconate. HexR acts as a transcriptional repressor in the absence of a specific effector, but the binding of KDPG to DNA-bound HexR causes protein dissociation and transcriptional activation [52]. Our results showed that knocking out the negative regulatory gene *hexR* of glucose metabolism could increase the content and titer of PHAs, and the coupling effect with *gcd* and *gltA* genes was more effective. Because there is no significant difference between CDW and PHA titers of QSRZ605 and QSRZ607, the QSRZ605 growth rate and glucose utilization were faster. Therefore, QSRZ605 was used as the starting strain for the next step of genetic engineering modification.

Thirdly, the expression of *gltB* and *phaD* encoded positive regulatory factors that were enhanced by strong promoter knock-in. GltR/GtrS, a two-component system, was found to participate in the transcriptional regulation of glucose catabolism. GtrS is a transmembrane sensor kinase that contains a periplasmic ligand binding domain. GtrS specifically recognizes 2-ketogluconate and 6-phosphogluconate, causing a modulation of its autokinase activity, resulting in efficient GtrS autophosphorylation and transphosphorylation to the GltR response regulator. GltR is a positive regulator that controls the transcription of *porB* gene, as well as the gtsABCD, edd/glk, and zwf/pgl/eda operon [26,53]. Periplasmic substrate-binding proteins (PBPs) recognize and deliver small molecules or ions into the cytoplasm via the cognate inner membrane ATP binding cassette (ABC) transport systems. PBPs were found to possess a number of additional functions, and there was increasing evidence for PBP-mediated activation of chemoreceptors and sensor kinases in gram-negative bacteria [54]. GltB (PP_5076), one of PBPs, interacts with GtrS and initiates the GtrS-GltR signaling cascade that slows *Pseudomonas* to respond to the presence of glucose [55]. In this study, two strongly expressed endogenous promoters were used to replace the original promoter to overexpress the *gltB* gene. The P_33_-initiated *gltB* overexpression mutant, QSZR609, had a CDW of 4.4 g/L, a PHA content of 57.3 wt%, an increase of 110.40% compared with QSZR6; and a PHAs titer of 2.52 g/L, an increase of 159.9% compared with QSZR6. The CDW and PHA production capacity of QSZR608 was not significantly different from that of the starting strain QSZR605, suggesting that the P_33_ promoter is more suitable for overexpression of the *gltB* gene. Therefore, the next step was to use QSZR609 as the starting strain for genetic engineering. The organization of the *pha* gene cluster in *P. putida* KT2440 comprises two main operons [31]. The first one encodes two mcl-PHA syntheses or polymerases, PhaC1 (PP_5003) and PhaC2 (pp_5005), a depolymerase (PhaZ, pp_5004, was disrupted in our previous study), and a transcriptional activator (PhaD, pp_5006). The second one, located adjacent to the first one but in the opposite direction, encodes the phasins *PhaF* (PP_5007) and *PhaI* (PP_5008), which are two major GAPs that play important structural and regulatory roles. The Pc1 and PI promoters were proposed as the major drivers of *pha* transcription (Figure 1). The *pha* cluster is transcriptionally activated by the binding of the transcriptional regulator PhaD to specific operator sequences at the P_c1_ and P_I_ promoters [25]. The original strain QSRZ6 used in this experiment successfully knocked out the PHA depolymerase gene *phaZ*, and theoretically, overexpression of the *phaD* gene can enhance the expression levels of PHA polymerase PhaC_1_ and C_2_. Our results showed that p*haD* overexpression did not increase the synthesis of PHAs but stimulated rapid cell growth and glucose utilization at high glucose concentrations. The reason may be that PHA precursor substances (R)-HA-CoA were not provided sufficiently to synthesize PHAs [25]. So, the further overexpress *phaG* (encoding 3-hydroxyacyl-ACP thioesterase) and *alkK* (encoding fatty acid-CoA ligase) genes for precursor synthesis should be performed to increase the mcl-PHA synthetic capacity.

Finally, the fermentation optimization for batch replenishment in shake flasks was carried out, and the mcl-PHA production capacity of mutants was evaluated by the optimized fermentation protocols. The results showed that the mcl-PHA content of QSRZ605 and QSRZ609 reached 58.34 and 59.11 wt%, respectively. *P. putida* KT2440 with decaying exponential feeding of nonanoic acid produced 109 g/L of dry biomass containing 63% PHA in a 5 L stirred tank bioreactor [56]. But the biosynthesis level of mcl-PHA with low-cost glucose as substrate is generally low, especially in shake flasks. The high mcl-PHA content (53.8 wt%) was obtained in the mutant with *upp* and *gcd*-inactive and *phaC* and *acoA* overexpression with glucose as substrate in a 5 L fermentor [20]. However, the QSRZ609 constructed in this study accumulated a higher mcl-PHA content using the substrate indirectly related carbon source under shaker culture conditions, which is the highest level reported so far. Thus, *P. putida* QSRZ609 was an excellent chassis for further genetic engineering targeting the de novo fatty acid biosynthesis pathway. To cut the mcl-PHA production cost, QSRZ609 and its derivative are expected to yield high mcl-PHA by the fermentation of cellulose hydrolyzed sugar.

## 5. Conclusions

The synthesis of mcl-PHA with indirectly related substrate glucose is an effective means to reduce cost and increase added value. In this study, strategies such as a truncated branching metabolic pathway, a glucose metabolic regulatory factor modification, and positive regulator overexpression of *pha* clusters were used to enhance mcl-PHA synthesis in *Pseudomonas putida* QSRZ6. The results showed that the multi-gene coupling strategy significantly increased the mcl-PHA content and the final titer. With optimized flask fermentation culture conditions, the highest level of mcl-PHA content (59.1 wt%) and titer (6.8 g/L) was obtained in the modified mutant QSRZ09 (Δ*gcd*Δ*hexR*P_33_::*gltB*) among the currently derived strains of *P. putida* KT2440. QSRZ09 has a great potential for the industrial production of mcl-PHA, and it can also be used as a chassis for further genetic modification. In future studies, the fatty acid de novo synthesis pathway needs to be further modified to increase the direct precursors (R-3-hydroxyacly-CoA) of mcl-PHA and optimization of fermentation conditions in the reactor with cellulose hydrolyzed sugar as a substrate to enhance the production of mcl-PHA.

## Figures and Tables

**Table 1:** Plasmids and strains in this study.

Strains	Description	References
*E. coli*		
DH5α	*supE44*, *ΔlacU169* (φ80 lacZΔM15), *hsdR17* (*rk-mk+*), *recA1*, *endA1*, *thi1*, *gyrA*, *relA*	Tiangen
S17-1	*RP4-2*(Km::Tn7,Tc::Mu-1), *pro-82*, *LAMpir*, *recA1*, *endA1*, *thiE1*, *hsdR17*, *creC510*	Tiangen
*P. putida*		
QSRZ6	KT2440*∆hsdR-∆phaZ*	Lab stock
QSRZ601	QSRZ6*∆gcd*	This study
QSRZ602	QSRZ6*∆gltA*	This study
QSRZ603	QSRZ6*∆gcd-∆gltA*	This study
QSRZ604	QSRZ6*∆hexR*	This study
QSRZ605	QSRZ6*∆gcd-∆hexR*	This study
QSRZ606	QSRZ6*∆gltA-∆hexR*	This study
QSRZ607	QSRZ6 *∆gcd-∆gltA-∆hexR*	This study
QSRZ608	QSRZ605::P_17_-*gltB*	This study
QSRZ609	QSRZ605::P_33_-*gltB*	This study
QSRZ610	QSRZ605::P_33_-gltB::P_33_-*phaD*	This study
Plasmids		
pK18*mobsacB*	Mobilizable vector, Km^R^, *sacB*	Lab stock
pK18-*∆gcd*	A derivative of pK18*mobsacB*, harboring homology arms of *gcd*	This study
pK18-*∆gltA*	A derivative of pK18*mobsacB*, harboring homology arms of *gltA*	This study
pK18-*∆hexR*	A derivative of pK18*mobsacB*, harboring homology arms of *hexR*	This study
pK18-P_17_-*gltB*	A derivative of pK18*mobsacB*, harboring P_17_-*gltB* fragment and homology arms	This study
pK18-P_33_-*gltB*	A derivative of pK18*mobsacB*, harboring P_33_-*gltB* fragment and homology arms	This study
pK18-P_33_-*phaD-∆hexR*	A derivative of pK18*mobsacB*, harboring P_33_-*gltB* fragment and homology arms of *hexR*	This study

**Table 2:** Structural composition of PHAs in the mutants.

Strains	3HHx (%)	3HO (%)	3HD (%)	3HDD (%)	3HTD (%)
QSRZ6	0.4 ± 0.1	11.4 ± 0.0	73.0 ± 0.7	7.3 ± 0.4	7.9 ± 0.4
QSRZ601	0.2 ± 0.4	10.0 ± 0.2	74.7 ± 1.2	7.4 ± 0.2	7.7 ± 0.5
QSRZ602	0.1 ± 0.1	9.6 ± 0.4	73.1 ± 0.1	9.0 ± 0.1	8.2 ± 0.3
QSRZ603	0.6 ± 0.1	12.8 ± 0.1	66.3 ± 0.7	10.1 ± 0.4	10.2 ± 0.1
QSRZ604	0.5 ± 0.4	11.9 ± 2.4	70.0 ± 6.8	8.5 ± 2.0	9.2 ± 2.2
QSRZ605	0.7 ± 0.2	13.3 ± 0.9	71.1 ± 3.4	7.6 ± 1.3	7.2 ± 1.1
QSRZ606	0.9 ± 0.3	13.2 ± 1.5	71.4 ± 4.8	7.0 ± 1.4	7.6 ± 1.6
QSRZ607	0.6 ± 0.3	13.3 ± 1.3	70.1 ± 4.8	8.1 ± 1.7	8.0 ± 1.5
QSRZ608	NA	14.5 ± 0.2	71.4 ± 0.3	6.7 ± 0.2	7.5 ± 0.1
QSRZ609	0.5 ± 0.4	14.8 ± 0.2	70.7 ± 0.3	6.6 ± 0.2	7.4 ± 0.1
QSRZ610	NA	10.4 ± 0.3	72.9 ± 0.5	9.0 ± 0.2	7.7 ± 0.2
QSRZ611	NA	14.2 ± 0.2	71.5 ± 0.5	7.6 ± 0.2	6.8 ± 0.2

## Data Availability

Data is contained within the article and Supplementary Material.

## Supplementary Materials

The following supporting information can be downloaded at: https://www.mdpi.com/article/10.3390/cimb46110761/s1, Figure S1: The construction and verification of the modified mutants; Figure S2: The amount of glucose remaining during the shake flask fermentation; Figure S3: Optimization of glucose concentration, pH, and feeding strategy; Table S1: All primers in this study; Table S2: The CDW, PHA content, and production of QSRZ607 with different glucose concentrations; Table S3: The CDW, PHA content, and production of QSRZ607 with two feeding strategies.
