# Supplementary material for: Modification of Glucose Metabolic Pathway to Enhance Polyhydroxyalkanoate Synthesis in Pseudomonas putida
Source: Curr Issues Mol Biol. 2024 Nov 10;46(11):12784–99. doi: 10.3390/cimb46110761 (PMC11592762; doi:10.3390/cimb46110761)
Supplement: Supplementary file 1 [file cimb-46-00761-s001.zip › cimb-3270871-supplementary.pdf]

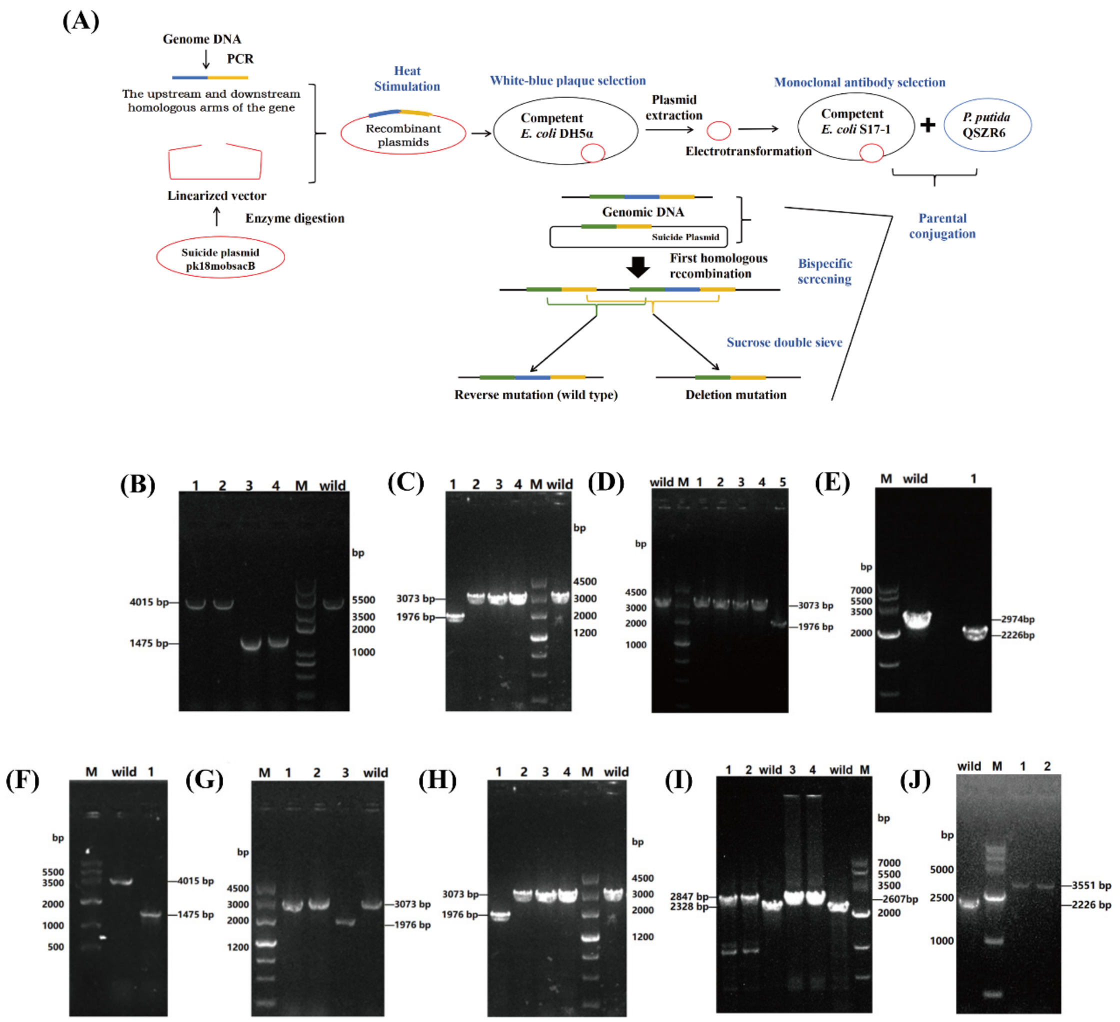

Figure S1. The construction and verification of the modified mutants. (A) Schematic diagram of the mutants construct process; (B) The verification of QSRZ601 by PCR with primers *gcd* F1 and *gcd* R2. Lane 1-2: negative clone, 3-4: positive clone; (C) The verification of QSRZ602 by PCR with primers *gltA* F1 and *gltA* R2. Lane 1: negative clone, 2-4: positive clone; (D) The verification of QSRZ603 by PCR with primers *gltA* F1 and *gltA* R2. Lane 1-4: negative clone, 5: positive clone; (E) The verification of QSRZ604 by PCR with primers *hexR* F1 and *hexR* R1. Lane 1： positive clone; (F) The verification of QSRZ605 by PCR with primers *gcd* F1 and *gcd* R2. Lane 1： positive clone; (G) The verification of QSRZ606 by PCR with primers *gltA* F1 and *gltA* R2. Lane 1-2: negative clone, lane 3: positive clone; (H) The verification of QSRZ607 by PCR with primers *gltA* F1 and *gltA* R2. Lane 1: Positive clone, lane 2-4: negative clone. (I) The verification of *gltB* overexpression mutants. lane 1-2: P. The PCR verification of putida QSRZ608 with primers *gltB* F1 and *gltB* R2; 3-4: The PCR verification of P. putida QSRZ609 with primers *gltB* F1 and *gltB* R2. (J): The verification of *phaD* overexpression mutants; Lane 1: The PCR

verification of QSRZ610 with primers *phaD* F1 and *phaD* R2; Lane 2: The PCR verification of QSRZ611 with primers *phaD* F1 and *phaD* R2.

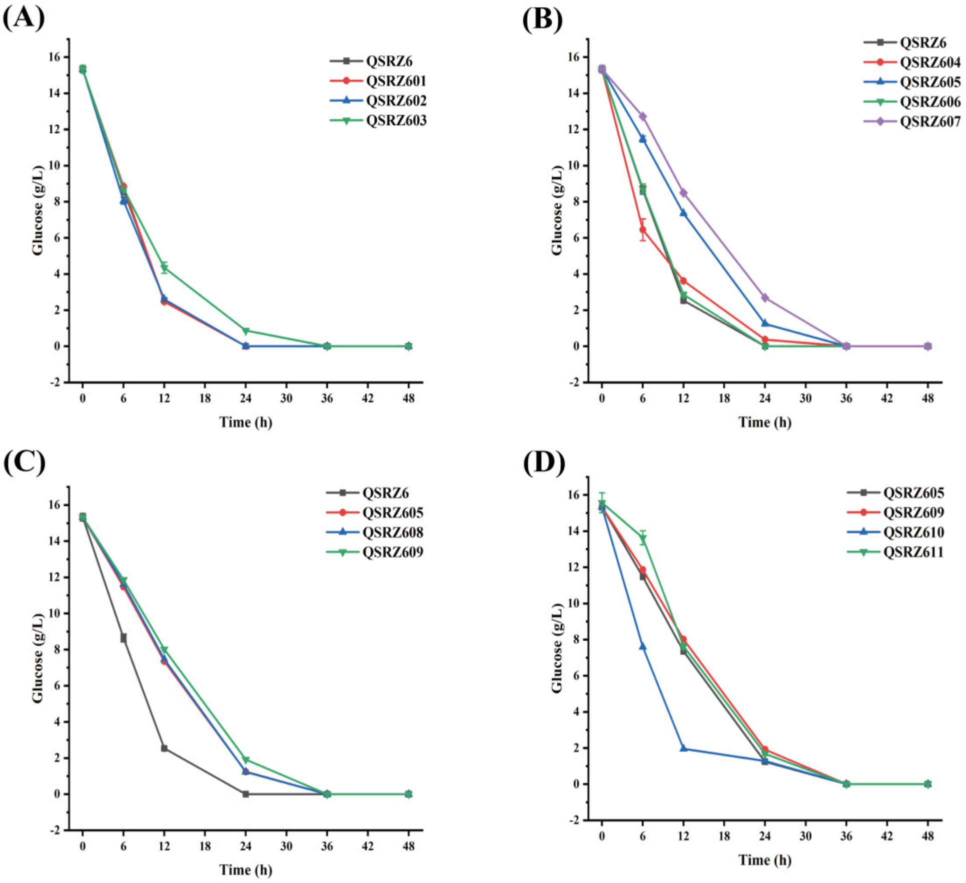

Figure S2 The amount of glucose remaining during the shake flask fermentation. (A) Residual sugar content of QSRZ6, QSRZ601, QSRZ602 and QSRZ603; (B): Residual sugar content of QSRZ6, QSRZ604, QSRZ605, QSRZ606 and QSRZ607; (C): Residual sugar content of QSRZ6, QSRZ605, QSRZ608 and QSRZ609; (D): Residual sugar content of QSRZ605, QSRZ609, QSRZ610 and QSRZ611.

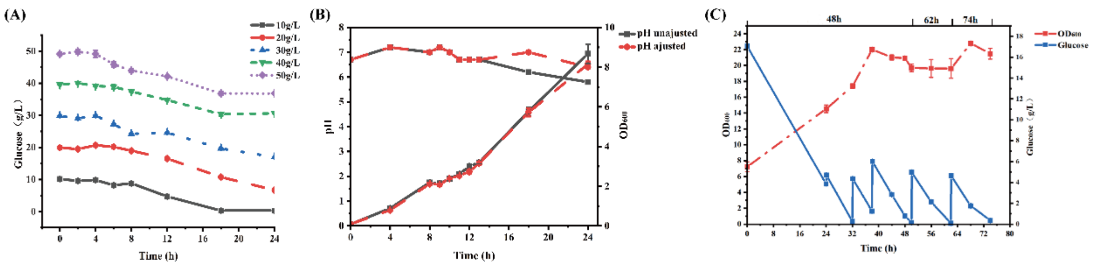

Figure S3 Optimization of glucose concentration, pH and feeding strategy. (A) Residual sugar content of QSRZ607 cultured in LBG medium with different

concentrations of glucose; (B) The cell growth of QSRZ607 in LBG medium with and without pH adjustment in culture; (C) the cell growth of QSRZ607 with a small amount of multiple feeding strategy.

Table S1 All primers in this study

**Primers**	**Sequences (5′~3′)**
gcd F1	agctcggtacccggggatccGCCATGCCGTAGGCTTTGAC
gcd R1	agaacctacgatgagCTGCTCCCGAACCATTGAAAC
gcd F2	tcaatggttcgggagCTCATCGTAGGTTCTCCGTC
gcd R2	cgacggccagtgccaagcttACTTCAACAGCTTCCCTTGC
gltA F1	agctcggtacccggggatccTTACCATACCCAAACTCCCACCAG
gltA R1	ccagggcgaagatcaCAGCGCCCTCGATGACCAACT
gltA F2	tcatcgagggcgctgTGATCTTCGCCCTGGCACGTA
gltA R2	cgacggccagtgccaagcttATCGCCCCGTCCATCACTTCT
hexR F1	agctcggtacccggggatccGCCAATCCATTTTCGGTTCC
hexR R1	accgtgcaaaaagccCCGACATCTACATGCCAATG
hexR F2	gcatgtagatgtcggGGCTTTTTGCACGGTCGTCT
hexR R2	cgacggccagtgccaagcttGGCGTTGGCGTTGTCTTCTT
gltB F1	agctcggtacccggggatccCTGTGGTCGTGGTTGCCGTT
gltB R1	GCAGGCTAGTCGAAAGCTAG
gltB FP17	tttcgactagcctgcGGGGATTCGCGTGGCAGAAGA
gltB RP17	tagaaaacctccttaCGCCCATAACCACATTCCAGAC
gltB P17-F2	atgtggttatgggcgtaaggaggttttctaATGAAAACAGGTCTGTACCATCCC
gltB R2	cgacggccagtgccaagcttCGATTTCCGAGGCGATGGTGA
gltB FP33	tttcgactagcctgcCGGTCCGACATGAGTATTCC
gltB RP33	tagaaaacctccttatGGTGTTGCCCTCACTTGTTG
gltB P33-F2	agtgagggcaacacctaaggaggttttctaATGAAAACAGGTCTGTACCATCCC
phaD hexR-F1	agctcggtacccggggatccGCCAATCCATTTTCGGTTCC
phaD hexR-R1	CCGACATCTACATGCCAATG
phaD FP33	gcatgtagatgtcggCGGTCCGACATGAGTATTCC
phaD RP33	tagaaaacctccttaGGTGTTGCCCTCACTTGTTG
phaD F	agtgagggcaacacctaaggaggttttctaATGAAAACCCGCGATCGTAT
PhaD R	accgtgcaaaaagccACCCTGATCTGATACCGCGT
phaD hexR-F2	GGCTTTTTGCACGGTCGTCT
phaD hexR-R2	cgacggccagtgccaagcttGGCGTTGGCGTTGTCTTCTT
phaD-qF	CCTGGAAATTGCCAACGAACTC
phaD-qR	ACAGCCAGTAATCCTCTGCGTC
16S-qF	CGGATCGCAGTCTGCAACTC
16S-qR	ACACCGTGGTAACCGTCCTC

Table S2 The CDW, PHAs content and production of QSRZ607 with different glucose concentrations

Glc initial content / Parameter	10 g/L	20 g/L	30 g/L	40 g/L	50 g/L
CDW(g/L)	7.35±0.05e	9.13±0.13d	10.67±0.16a	10.08±0.03b	9.69±0.09c
PHAs(wt%)	46.76±0.98d	51.08±1.44c	56.20±1.17a	55.93±1.80ab	53.18±1.46bc
PHAs(g/L)	3.44±0.07e	4.66±0.13d	6.00±0.13a	5.63±0.18b	5.15±0.14c

Note: Different lowercase letters in the same industry indicate significant differences among different mutant strains (p<0.05).

Table S3 The CDW, PHA content and production of QSRZ607 with two feeding strategy

Feeding strategy	Single feeding to 10g/L	Multiple feeding to 4-6g/L		
Time(h) / Parameter	48	48	62	74
CDW(g/L)	11.58±0.03c	12.64±0.11b	12.97±0.03a	13.00±0.01a
PHAs(wt%)	58.34±2.74a	54.22±0.33b	56.00±2.16b	55.45±2.70b
PHAs(g/L)	6.75±0.32	6.85±0.09	7.26±0.27	7.20±0.35

Note: Different lowercase letters in the same industry indicate significant differences among different mutant strains (p<0.05).
